# Meetings of Societies

**Published:** 1896-12

**Authors:** 


### MEETINGS OF SOCIETIES.

### Bristol Medico-Chirurgical Society.

*Annual Meeting, October 14th*, 1896.

Mr. ARTHUR W. PRICHARD, President, in the Chair.

After a vote of thanks to the retiring President (Mr. Arthur W. Prichard) had been unanimously passed, Dr. A. E. Aust Lawrence (the President-elect) took the Chair, and read his introductory address (see page 289), for which a cordial vote of thanks was given him. The Honorary Secretary (Mr. J. Paul Bush), the Editorial Secretary (Dr. Bertram M. H. Rogers), and the Honorary Librarian (Mr. L. M. Griffiths) read their annual reports, which were adopted. The following officers were chosen for the ensuing year:—President-elect; Dr. J. E. Shaw: Honorary Secretary; Mr. J. Paul Bush: Honorary Librarian; Mr. L. M. Griffiths: Members of Committee; Dr. J. Michell Clarke, Mr. J. Dacre, Mr. W. H. Harsant, Dr. Bertram M. H. Rogers, Mr. G. Munro Smith, and Dr. H. Waldo: Representatives of the Society on the Committee of the Bristol Medical Library; Mr. L. M. Griffiths, Dr. G. Parker, and Dr. J. Swain.

*November* 11*th*, 1896.

Dr. A. E. Aust Lawrence, President, in the Chair.

Dr. Barclay J. Baron showed a case of a healthy farm labourer, 39 years of age, who came to him with a history of acute œdematous laryngitis, for which laryngotomy had been performed. On admission to the Bristol General Hospital the whole of the larynx was found to be in a state of acute inflammation, the inflamed vocal cords lying in apposition in the middle line. So long as he had his laryngotomy tube in his larynx the condition did not in any way alter. Dr. Baron recommended him to submit to tracheotomy, the laryngotomy tube being then removed. Very soon after this the inflammation subsided, and first one vocal cord and then the other left the middle line, and a web occupying the anterior three-fourths of the glottis was visible. This Dr. Baron divided with a cutting dilator several times, and passed Schrötter's bougies; and now the patient is left with a piece of web-tissue uniting the under surface of the anterior sixth of the vocal cords. His voice is good, and he can do all that a labourer has to do, and so nothing further in the way of operation will be done. The two points of interest in the case are: (1) The great rarity of ulceration of the inner edges of both vocal cords occurring as a result of simple non-syphilitic inflammation of the larynx, and this permitting of the formation of the web seen. (2) The laryngotomy tube was responsible for keeping up the laryngeal inflammation, and only when it was removed did resolution of this occur. Dr. Baron considers that where there is acute œdematous inflammation of the larynx, and an opening into the windpipe is necessary, tracheotomy should be performed and not laryngotomy.—Dr. P. Watson Williams had met with two cases of web-formation extending between the vocal cords. One was congenital in origin, the second being the result of syphilitic disease.

Dr. P. Watson Williams showed (1) a case of tuberculous disease of the larynx very greatly improved by curettement and the local application of lactic acid and of pure guaiacol; (2) a case of suppurating perichondritis of the thyroid cartilage, with resulting laryngeal stenosis; (3) a case in which an almost complete nasal stenosis resulted from persistence of the normal fœtal occlusion of the nasal passage.

Mr. C. A. Morton showed a man, aged 55, from whom, on June 12th, he had removed the right half of the tongue and floor of the mouth, and the right tonsil, for epithelioma, by external pharyngotomy. There had been epitheliomatous ulceration involving the back and right side of the tongue and floor of the mouth, and the right tonsil, and the patient had only been able to swallow fluids, and those only with much distress. The lingual artery was first tied on the left side, and then tracheotomy performed and a Hahn's tube introduced into the trachea. An incision was then made on the right side of the neck, as in Kocher's method of excising the tongue; but the jaw was also divided at the junction of the body and ascending ramus, and thus free access gained to the tonsillar region. A portion of the external pterygoid muscle had to be removed with the tonsil. The lingual artery was tied just as it left the external carotid, and other arteries as they were cut, the division of the jaw giving sufficient room to deal with all bleeding vessels. The external carotid artery was not ligatured, as in several recorded cases secondary hemorrhage has followed the ligation of the artery in what, on account of the free communication with the mouth, must be a septic wound. The tongue was found to be infiltrated with growth almost down to the hyoid bone, and had to be divided close to that structure, thus fully exposing the epiglottis. The wound was not closed, but was plugged with gauze; and the patient fed by a tube left in the stomach, with the end protruding through the wound in the neck. He rapidly gained flesh from the constant feeding through the tube, and can now swallow mince-meat and other kinds of food; the opening in the neck has been quite closed for some months, and he has returned to work as a fireman on an engine. There is no sign of any recurrence so far, *i.e.* five months after the operation.—Dr. J. LACY FIRTH mentioned a case in which he had recently removed an upper jaw for epithelioma; the glands having been removed a week earlier by Mr. Barclay, who, having to leave home, had left the case in his charge. There was a very rapid recurrence in the neck; and this was like the event of a case quoted by Mr. Watson Cheyne, in which he also did the operation in two stages. Operating in two stages was therefore bad practice.—Mr. W. M. BARCLAY referred to the case, mentioned by Dr. Firth, in which he had done the original operation of removal of the secondary glands. It was intended that the entire operation should be concluded at once; but after the glandular extirpation it was thought wiser to defer the removal of the growth in the mouth. The point to which he wished to draw attention was as to the cause of the very rapid recurrence in the neck. He recalled a suggestion of Mr. Watson Cheyne's, that these rapid recurrences might result from an actual implantation of cancerous material in the wound by a rupture of the infected glands, rather than from the cut lymphatic vessels leading from the glands to the primary growth. If this theory be ever correct, a very important practical point is involved; viz., that in all operations for removal of carcinomatous glands every endeavour should be made to remove them *en masse*, without opening their capsules proper or adventitious.—Mr. MORTON said the point raised by Dr. Firth and Mr. Barclay was one of great interest. Mr. Watson Cheyne hoped by first removing the glands and tying the external carotid artery he would get the vessel so satisfactorily closed in the aseptic wound that secondary hemorrhage would not occur; but he found that the wound would become infected with epitheliomatous material from the primary growth through the cut lymphatic vessels, and he thought that early recurrence in one of his cases had been due to this.

Two patients were sent by Dr. H. WALDO. The first was a case of adenoma sebaceum in a girl aged 16, who had had much local treatment, resulting in considerable scarring. She has been advised to become an in-patient at the Bristol Royal Infirmary, in order that the remaining diseased surface may be scraped. The second case was one of alopecia areata in a girl aged 12, who began to lose her hair a short time after an attack of influenza about two years ago. In a few weeks it all came off. There was not a smoothness of the billiard ball character, as there was an extremely soft down in very small quantity. Treatment by Stoker's cap was begun on August 8th, 1896. It was kept full of oxygen, and now the scalp is nearly covered with hair, which is thin and silky, and an inch or more in length.

Dr. J. MICHELL CLARKE read an account and showed microscopic specimens and lantern slides of two cases of congenital syphilitic cirrhosis in infants, illustrating the progress of the disease. He also showed lantern slides and specimens of a cavity in the cord (syringomyelia). The cavity was situated behind the posterior commissure of the cord, and was surrounded by embryonic tissue. It was about 1½ inches in length and about ⅛ inch in transverse diameter. There were no symptoms during life. The patient was a man who died from pernicious anæmia, and whose case was reported in the September number of the *Bristol Medico-Chirurgical Journal* (p. 232). A brief account was given of the mode of origin of these cavities in the cord in syringomyelia, and of the symptoms which characterize the affection. Dr. Clarke also showed sections of the cord from another case of pernicious anæmia, in which there was sclerosis of the columns of Goll, of part of the columns of Burdach, and of the crossed pyramidal tracts throughout the whole extent of the cord; and remarked on the not infrequent occurrence of such degenerations in pernicious anæmia.

J. PAUL BUSH, *Hon. Sec.*

### Newport Medical Society.

*Annual Meeting, October 7th,* 1896.

Dr. A. GARROD THOMAS in the Chair.

The annual report was read and adopted, and the following elected officers for the ensuing session :—President ; Mr. G. A. Davies : Vice-President ; Mr. H. M. Brewer : Honorary Secretary ; Mr. W. Basset. The two vacancies on the Committee were filled by the election of Messrs. A. G. Thomas and H. E. Williams.

Mr. O. E. B. MARSH showed two cases of lateral dislocation of the elbow.

Dr. D. M. BEDDOE showed two cases of congenital disease of the pulmonary artery.

Mr. W. BASSET showed a case of congenital fracture of the leg in a child six years of age.

W. BASSET, *Hon. Sec.*

